# Heat Adaptation among the Elderly in Spain (1983–2018)

**DOI:** 10.3390/ijerph20021314

**Published:** 2023-01-11

**Authors:** Miguel Ángel Navas-Martín, José Antonio López-Bueno, María Soledad Ascaso-Sánchez, Fernando Follos, José Manuel Vellón, Isidro Juan Mirón, María Yolanda Luna, Gerardo Sánchez-Martínez, Cristina Linares, Julio Díaz

**Affiliations:** 1 National School of Public Health, Carlos III Institute of Health, 28029 Madrid, Spain; 2 Doctorate Program in Biomedical Sciences and Public Health, National University of Distance Education, 28015 Madrid, Spain; 3 Tdot Soluciones Sostenibles, SL., Ferrol, 15401 A Coruña, Spain; 4 Regional Health Authority of Castile La Mancha, 45500 Torrijos, Spain; 5 State Meteorological Agency, 28071 Madrid, Spain; 6 The UNEP DTU Partnership, 2100 Copenhagen, Denmark

**Keywords:** adaptation, MMT, age, prevention plan, mortality, health

## Abstract

The capacity for adaptation to climate change is limited, and the elderly rank high among the most exposed population groups. To date, few studies have addressed the issue of heat adaptation, and little is known about the long-term effects of exposure to heat. One indicator that allows the ascertainment of a population’s level of adaptation to heat is the minimum mortality temperature (MMT), which links temperature and daily mortality. The aim of this study was to ascertain, firstly, adaptation to heat among persons aged ≥ 65 years across the period 1983 to 2018 through analysis of the MMT; and secondly, the trend in such adaptation to heat over time with respect to the total population. A retrospective longitudinal ecological time series study was conducted, using data on daily mortality and maximum daily temperature across the study period. Over time, the MMT was highest among elderly people, with a value of 28.6 °C (95%CI 28.3–28.9) versus 28.2 °C (95%CI 27.83–28.51) for the total population, though this difference was not statistically significant. A total of 62% of Spanish provinces included populations of elderly people that had adapted to heat during the study period. In general, elderly persons’ level of adaptation registered an average value of 0.11 (°C/decade).

## 1. Introduction

The Intergovernmental Panel on Climate Change (IPCC) predicts that if current human activity continues, there will be a 1.5 °C increase in global warming [1]. This rise in temperature is expected to lead to increased mortality and morbidity risks [2]. Populations with high vulnerability and exposure to heat, such as the elderly, chronic patients, and children [2,3,4], must prioritize adaptation measures [5]. However, there is little information on how the population is adapting to climate change [6].

An indicator that makes it possible to measure whether a given population is adapting to heat is the time trend in the minimum mortality temperature (MMT) [6,7,8]. The MMT is characterised by linking temperature and daily mortality [8,9,10,11], this association usually has a shape depicted graphically as a U-, V- or J-shaped curve [7,12,13,14,15,16].

Effective heat health action plans that are adapted to local conditions must be implemented by all nations, local communities, and institutions. To minimize the impact of heat, some governments have implemented action plans to address heat-related health [17]. These action plans include alert systems to reduce vulnerability to heat [2]. In Spain, the National Heat Wave Prevention Plan (Plan Nacional de Actuaciones Preventivas de los Efectos del Exceso de Temperatures Sobre la Salud) was created in 2004 as an instrument to combat the effects of high temperatures on health [18].

The best way of establishing that an action plan is working is to ascertain whether heat-related mortality is decreasing. Yet the way of assessing mortality differs according to the place and type of population involved, which, coupled with the use of different methods, hinders comparison and interpretation of results [9,19]. Use of the time trend in the MMT is thus key to assessing the effectiveness of such plans [9].

There are few studies that analyse the population impact of climate change adaptation to extreme heat [20]. Similarly, little is known about human beings’ capacity for adaptation to long-term exposure to heat [6]. Furthermore, ranking high among the groups most vulnerable to the effects of heat are the elderly [3], bearing in mind that the MMT could be altered by further population ageing [21].

Ageing affects people’s thermal response, in that they lose the capacity for thermoregulation, sweating and sensation of thirst. As ageing advances, vulnerability rises, becoming greatest after 65 years of age [6,22].

This study thus sought to ascertain, firstly, elderly persons’ (age ≥ 65 years) adaptation to heat across the period 1983–2018, through analysis of the MMT, and secondly, the differences with respect to the total population. A retrospective longitudinal ecological time series study was conducted, using data on daily mortality and maximum daily temperature during the study period.

## 2. Materials and Methods

We conducted a retrospective longitudinal ecological time series study on the population aged ≥ 65 years in Spain’s provinces, across the period 1983–2018. 

Daily mortality was calculated using microdata furnished by the National Statistics Institute (Instituto Nacional de Estadística/INE) under an institutional agreement. We used daily mortality data, coded by all natural causes of death, covering elderly persons in towns of over 10,000 inhabitants, broken down by province, during the study period.

Temperature was calculated using data registered at reference observatories in each province across the study period and supplied by the State Meteorological Agency (Agencia Estatal de Meteorología/AEMET).

The following were discarded: any record that lacked data on mortality and/or temperature; and any year of any given provincial time series for which more than 10% of the annual records were missing.

### 2.1. Calculation of MMT

The MMT was calculated using the deterministic methodology proposed by authors [9,10,11]. To this end, average mortality, grouped into intervals of two degrees Celsius, was calculated by province for each year of the time series for the elderly population. We then fitted a cubic or quadratic regression model of mortality aggregated with temperature, selecting the MMT values that were significant (*p*-value < 0.05). In cases where the MMT was not significant, MMT estimates were made to complement the calculations, using the method applied by Navas-Martín et al. [11]. This new methodology makes it possible to complement the calculation of MMTs by using the average of the maximum daily temperatures observed under the 5th percentile of mortality. Lastly, in any case where MMTs were not obtained by estimation, these were discarded. MMTs were calculated for each year and province.

**Figure 1:**
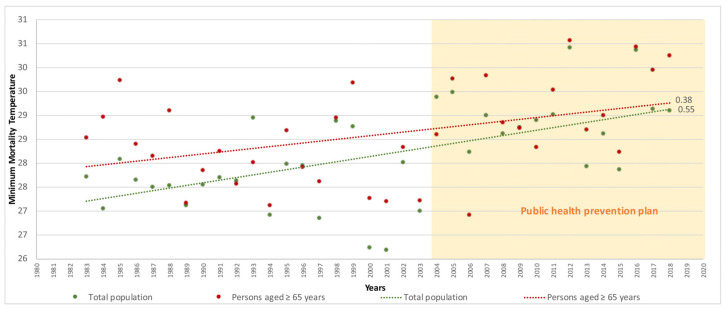
Trend in minimum mortality temperature (MMT) by year for age groups ≥ 65 years and the total population in Spain (1983–2018).

**Figure 2:**
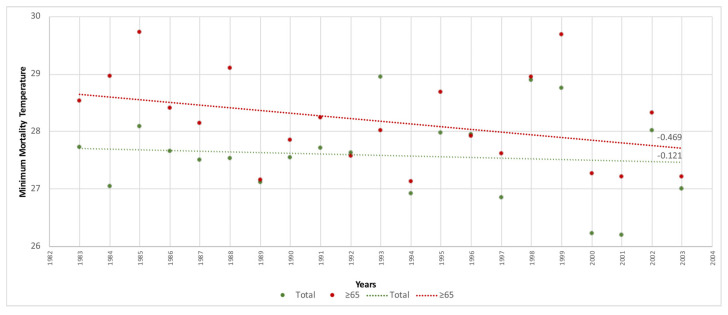
Trend in minimum mortality temperature (MMT) by year for age groups ≥ 65 years and the total population in Spain (1983–2003).

**Figure 3:**
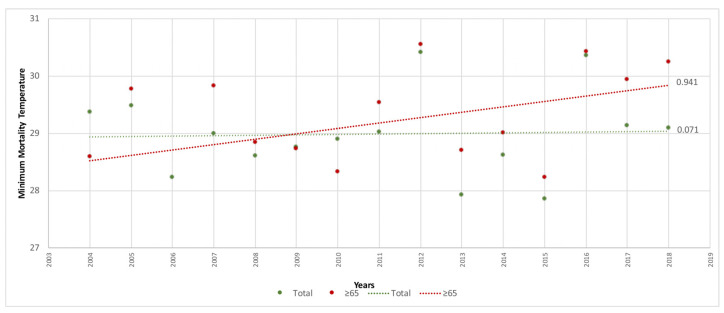
Trend in minimum mortality temperature (MMT) by year for age groups ≥ 65 years and the total population in Spain (2004–2018).

To ascertain the time trend in MMT for the elderly versus the total population according to a previous study [10], the linear regressions of the MMT were calculated for the entire study period (Figure 1), for the period 1983–2003 (Figure 2) which corresponded to the study years when there was no public health prevention plan, and for the period 2004–2018 (Figure 3), which was when the first public health sponsored heat wave prevention plan was introduced in Spain to combat the effects of excess temperatures on health.

### 2.2. Determination of Heat Adaptation Levels

To determine the elderly population’s level of adaptation, we calculated the variations in maximum daily temperature (TMAX Rise) and (MMT Variation) expressed in °C/decade, by fitting linear regression models.

The adaptation level was then established as the difference between MMT Variation and TMAX Rise. 

This made it possible to obtain (Table 1) the variations in maximum daily temperature, MMT, and the adaptation levels of the elderly population age group. 

### 2.3. Data Analysis

Data processing was performed using the R version 4.0.2, IBM SPSS Statistics version 28, STATA BE-Basic Edition version 17, and Excel 2019 (with the Power Query add-in) computer software programs.

## 3. Results

A total of 92.4% (*n* = 1596) of MMTs were obtained, with only 7.6% (*n* = 132) of these being discarded. Of the total MMTs calculated, 8.6% (*n* = 149) corresponded to a quadratic fit, 12.6% (*n* = 218) to an estimation-based fit, and 71.1% (*n* = 1229) to a cubic fit. 

With respect to the time trend in the MMT for the population aged ≥ 65 years versus the total population, the MMT of the elderly population group was higher in all periods. Across the entire study period (Figure 1), the elderly had a mean value of 28.6 °C (95%CI 28.3–28.9) while the total population had a mean value of 28.2 °C (95%CI 27.83–28.51), though this difference was not statistically significant. During the years in which there were no prevention plans (Figure 2), elderly persons had a mean value of 28.2 °C (95%CI 27.8–28.5) versus 27.6 °C (95%CI 27.2–27.9) for the total population. In the years when plans were already being implemented (Figure 3), the elderly population had a mean value of 29.2 °C (95%CI 28.6–29.7) versus 29 °C (95%CI 28.6–29.4) for the total population. Here too, the differences found were not statistically significant.

In terms of the MMT trend by year, this was higher in the total population than in the elderly population group, with figures of 0.55 (°C/decade) versus 0.38 (°C/decade) respectively. During the period when there were no prevention plans, and despite the trend being downward in both groups, the MMT was again higher in the total population, with −0.12 (°C/decade) versus −0.47 (°C/decade) in the elderly population. In contrast, this trend changed during the period when prevention plans already existed in Spain, with 0.94 (°C/decade) among the elderly versus 0.07 (°C/decade) among the total population.

**Figure 4:**
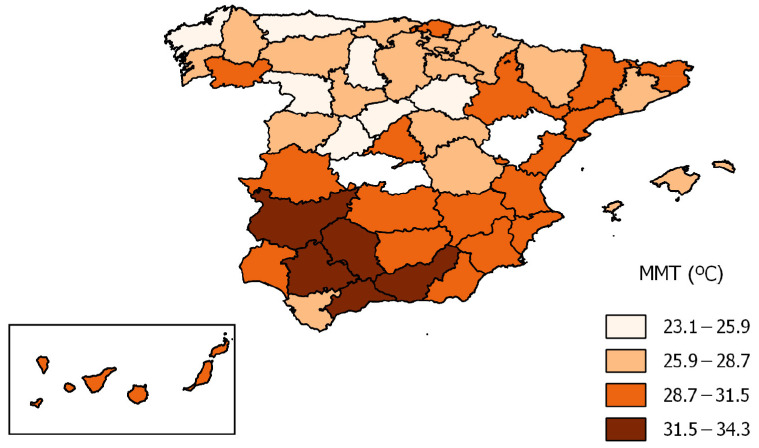
MMT by province for age groups ≥ 65 years in Spain (1983–2018).

Taking the MMT (Figure 4) by province, the mean value (Table 1) of the elderly population was 28.4 °C (95%CI 27.7–29.2) for Spain as a whole, with Avila being the province that registered the lowest value (23.1 °C) and Cordoba the highest value (34.3 °C). The average level of variation in MMT for all provinces was 0.45 (°C/decade).

**Figure 5:**
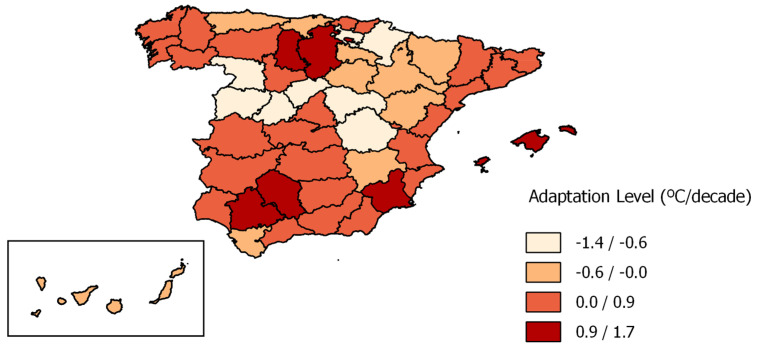
Adaptation level by province for age groups ≥ 65 years in Spain (1983–2018).

Analysis of the elderly population’s adaptation level by province (Figure 5) showed that 62% of provinces (*n* = 31) adapted versus 38% (*n* = 19) which did not. The average adaptation level was 0.11 (°C/decade).

## 4. Discussion

There is little evidence as to how the population will adapt or is adapting to heat due to climate change [6,20]. This is coupled with the fact that vulnerability to climate change comes about in different ways, causing different responses in the population’s adaptation [23].

This study analysed heat adaptation with reference to the MMT of the population aged ≥ 65 years in Spain across the period 1983–2018. It is only logical that the best way of ascertaining how the MMT will affect a given population’s adaptation to heat in the future, is by studying its effects in the past.

### 4.1. Vulnerability to Heat in the Elderly

The elderly population’s average MMT value was found to be 0.4 °C higher than the general population’s. If the MMT is used as an indicator of heat vulnerability, it appears that the population aged ≥ 65 years is less vulnerable to heat than the general population, although no statistically significant difference was found. This finding is consistent with a recent study that found that the 18–44-year-old age group had a higher percentage risk of heat-related mortality than other age groups, and that this risk had increased over time [24]. It also agrees with another study on several Spanish cities, which reported that mortality among persons aged ≥ 70 years was similar to mortality in the total population [25].

Although elderly persons are more susceptible to non-optimal temperatures than the young and middle-aged adult populations [26], there are a range of factors which could account for the fact that the elderly population has reduced its vulnerability to heat. Improvements in dwellings, infrastructures, use of air-conditioning equipment, heightened awareness of and sensitivity to prevention campaigns [6,27] and behavioural changes, such as opening and closing windows to improve airflow and change the indoor temperature [28], have meant that older persons may be more resistant to heat.

Similarly, it was observed that during the years when there were no plans, the difference in the MMT between the elderly and total populations was 0.6 °C, whereas during the years when plans were implemented, the gap closed, with the difference in their respective MMTs being only 0.2 °C. This may be due to the fact that not only were the plans effective for the population as a whole, but that with the passage of years and thanks to previous campaigns, the elderly population has also become more successful at internalising the heat culture.

At a provincial level, the average variation in the MMT of the elderly population was 0.45 (°C/decade), with this being similar to that obtained for general population, i.e., 0.41 (°C/decade) according to a previous study [10]. The fact that in Spain the average proportion of persons aged ≥ 65 years across the study period was 40% of the country’s population, with this figure being 28.89% in 1983 and 53.43% in 2018 [29], added to the fact that the mortality rate was 27 times higher in the elderly than in the young and middle-aged populations, means that the population aged ≥ 65 years may be over-represented in mortality [30] and, by extension, in its relationship with temperature. 

With respect to the trend in MMT over time in persons aged ≥ 65 years and in the total population, this was higher in the total population group than in the elderly population, though during the period when there were no prevention plans, the trend was downward in the elderly population group. This finding suggests, as do others studies in various countries, as well as a study conducted in Spain [31], that prevention plans contribute to the decrease in heat-related mortality [32,33,34]. 

### 4.2. Adaptation to Heat in the Elderly Population

As regards the elderly population’s level of adaptation, most of the provinces showed adaptation, and in general, the elderly Spanish population has adapted to heat. This is in line with the trend in other countries in the region, such as France, where with the passage of time, the general population has shown adaptation to heat due to global warming [35]. The effects of heat have declined over time [36], and in the ≥ 65 age group in particular [27].

The elderly population Is not only more vulnerable to heat, it also has a lower response in terms of sweating and hydration needs and is also more physically debilitated. In contrast, however, it has a greater capacity for acclimatisation to heat. This could be accounted for by an enhancement in living conditions, such as improved health services, socio-economic factors, adapted dwellings, use of air-conditioning equipment, heightened awareness of the risks, and a greater receptive capacity with regard to heat prevention campaigns [6,27,36,37].

This highlights the need for studies into the possible links between global warming and population ageing, in order to improve public health policies and the planning of heat adaptation strategies focused on elderly populations [37].

Likewise, there is a need for more studies to explore the relationship between mortality and temperature in localized [5,38,39], and so allow for more in-depth studies to be undertaken for eventual application to public health prevention plans.

## 5. Limitations

This study has several limitations, due firstly to its being the type of ecological study in which the results cannot be interpreted at an individual level [40], and secondly, due to the lack of quality of certain data from some provinces or the absence of other data, such as the air pollution data that could not be included in the study.

Lastly, there is no universal methodology for calculating MMT. Although there is a clear relationship between the temperature-mortality association, with this being graphically depicted as a U-, V- or J-shaped curve [7,12,13,14,15,16], there are nonetheless differences in its analysis, with some researchers using the absolute or relative MMT value, or both [21].

While many studies use distributed lag non-linear models (DLNMs) [41], their use is not free of bias [42]. Similarly, there are differences in criteria for obtaining MMTs: for the calculation of risk, some studies use percentile values above the 50th percentile [43], the 84th percentile [44], the 95th percentile [45,46], or the 99th percentile [42,47]. Other authors do not include lag [48] or include two days [49] or twenty-one days [26] in their studies.

## 6. Conclusions

Although the Spanish provincial population aged ≥ 65 years showed adaptation to heat, heterogeneities were found among the various provinces, highlighting the fact that in a given country vulnerability to ambient temperature varies, and that its relationship with the process of adaptation is thus influenced by the geographical, climatic and socio-economic characteristics of each particular area.

## Figures and Tables

**Table 1:** Relationship of variables, by province, between minimum mortality temperature (MMT) in persons aged 65 years or over, maximum daily temperature (TMAX), mean TMAX, increase by decade in TMAX, variations in MMT and adaptation levels. * *p*-value < 0.05.

Code	Name	MMT	Mean (°C)	TMAX Rise (°C/Decade)	MMT Variation (°C/Decade)	Adaptation Level (MMT Variation-Tmax Rise)
1	Araba	28.2	17.4	0.459	−0.299	−0.758
2	Albacete	30.4	21	0.509	0.337	−0.172
3	Alicante	30.2	23.5	0.190	0.817	0.627 *
4	Almería	31.3	23.4	−0.070	0.531	0.601
5	Avila	23.1	17.2	0.394	−0.737	−1.131
6	Badajoz	32.8	24	0.286	0.490	0.204
7	Balearic Isles	28.6	22	0.330	1.449	1.119 *
8	Barcelona	26.9	20.6	0.414	0.483	0.069 *
9	Burgos	27.4	16.8	0.372	1.611	1.239
10	Cáceres	29.7	22.1	0.336	0.623	0.287
11	Cadiz	28.2	21.7	0.287	−0.230	−0.517
12	Castellón	29.9	22.5	0.370	0.757	0.387
13	Ciudad Real	29.5	22	0.267	0.341	0.074
14	Cordoba	34.3	25.4	0.332	1.887	1.555 *
15	Corunna	24.7	18	0.351	0.832	0.481
16	Cuenca	26.1	19.6	0.617	−0.245	−0.862
17	Girona	29.5	21.1	0.656	0.980	0.324
18	Granada	31.7	22.6	0.416	1.018	0.602 *
19	Guadalajara	26.4	20.5	0.367	−1.054	−1.421
20	Gipuzkoa	26.3	16.6	0.244	0.328	0.084
21	Huelva	30.4	24.1	0.322	0.916	0.594
22	Huesca	27.8	19.8	0.489	0.442	−0.047
23	Jaén	30.2	21.8	0.516	1.299	0.783 *
24	León	26.4	16.9	0.243	0.516	0.273
25	Lleida	30.3	21.7	0.264	0.499	0.235
26	Rioja, La	27.5	19.8	0.416	0.091	−0.325
27	Lugo	27.9	17.8	0.189	1.060	0.871
28	Madrid	29.2	20.2	0.394	0.816	0.422 *
29	Malaga	31.5	23.5	0.320	0.327	0.007
30	Murcia	30.3	22.4	0.172	1.510	1.338 *
31	Navarre	27.2	18.6	0.442	−0.344	−0.786
32	Ourense	31.4	21.6	0.457	0.973	0.516
33	Asturias	25.3	17.5	0.184	−0.047	−0.231
34	Palencia	24.0	16.8	0.286	1.953	1.667
35	Palmas, Las	29.4	24.3	0.128	−0.495	−0.623
36	Pontevedra	26.4	19.1	0.099	0.455	0.356
37	Salamanca	27.4	19	0.613	−0.442	−1.055
38	S.C. Tenerife	30.1	24.7	0.225	−0.342	−0.567
39	Cantabria	26.6	18.7	0.277	−0.175	−0.452
40	Segovia	23.8	18.1	0.298	−0.450	−0.748
41	Seville	34.0	25.6	0.310	1.425	1.115 *
42	Soria	24.3	17.3	0.280	0.035	−0.245
43	Tarragona	28.8	21.3	0.380	0.484	0.104
44	Teruel	23.8	19.9	0.420	−0.122 *	−0.542
45	Toledo	30.2	22.4	0.412	1.197	0.785 *
46	Valencia	31.0	22.9	0.313	0.359	0.046
47	Valladolid	26.5	17.8	0.186	0.225 *	0.039
48	Bizkaia	29.1	19.7	0.062	0.210	0.148
49	Zamora	25.9	19.2	0.491	−0.194	−0.685
50	Zaragoza	30.0	21.3	0.472	0.377	−0.095
	(Spain)	28.4	20.6	0.34	0.45	0.11

## Data Availability

Data sharing not applicable.

## References

[1] (2022). IPCC Climate Change 2022: Impacts, Adaptation and Vulnerability—IPCC. https://www.ipcc.ch/report/ar6/wg2/.

[2] Ebi K.L., Capon A., Berry P., Broderick C., de Dear R., Havenith G., Honda Y., Kovats R.S., Ma W., Malik A. (2021). Hot weather and heat extremes: Health risks. Lancet.

[3] Benmarhnia T., Deguen S., Kaufman J.S., Smargiassi A. (2015). Vulnerability to heat-related mortality: A systematic review, meta-analysis, and meta-regression analysis. Epidemiology.

[4] Yang J., Zhou M., Ren Z., Li M., Wang B., Liu D.L., Ou C.Q., Yin P., Sun J., Tong S. (2021). Projecting heat-related excess mortality under climate change scenarios in China. Nat. Commun..

[5] Estoque R.C., Ooba M., Seposo X.T., Togawa T., Hijioka Y., Takahashi K., Nakamura S. (2020). Heat health risk assessment in Philippine cities using remotely sensed data and social-ecological indicators. Nat. Commun..

[6] Folkerts M.A., Bröde P., Botzen W.J.W., Martinius M.L., Gerrett N., Harmsen C.N., Daanen H.A.M. (2020). Long Term Adaptation to Heat Stress: Shifts in the Minimum Mortality Temperature in the Netherlands. Front. Physiol..

[7] Yin Q., Wang J., Ren Z., Li J., Guo Y. (2019). Mapping the increased minimum mortality temperatures in the context of global climate change. Nat. Commun..

[8] López-Bueno J.A., Díaz J., Follos F., Vellón J.M., Navas M.A., Culqui D., Luna M.Y., Sánchez-Martínez G., Linares C. (2021). Evolution of the threshold temperature definition of a heat wave vs. evolution of the minimum mortality temperature: A case study in Spain during the 1983–2018 period. Environ. Sci. Eur..

[9] Follos F., Linares C., Vellón J.M., López-Bueno J.A., Luna M.Y., Sánchez-Martínez G., Díaz J. (2020). The evolution of minimum mortality temperatures as an indicator of heat adaptation: The cases of Madrid and Seville (Spain). Sci. Total Environ..

[10] Follos F., Linares C., López-Bueno J.A., Navas M.A., Culqui D., Vellón J.M., Luna M.Y., Sánchez-Martínez G., Díaz J. (2021). Evolution of the minimum mortality temperature (1983–2018): Is Spain adapting to heat?. Sci. Total Environ..

[11] Navas-Martín M.Á., López-Bueno J.A., Ascaso-Sánchez M.S., Sarmiento-Suárez R., Follos F., Vellón J.M., Mirón I.J., Luna M.Y., Sánchez-Martínez G., Culqui D. (2022). Gender differences in adaptation to heat in Spain (1983–2018). Environ. Res..

[12] Arbuthnott K., Hajat S., Heaviside C., Vardoulakis S. (2018). What is cold-related mortality? A multi-disciplinary perspective to inform climate change impact assessments. Environ. Int..

[13] Rai M., Breitner S., Wolf K., Peters A., Schneider A., Chen K. (2019). Impact of climate and population change on temperature-related mortality burden in Bavaria, Germany. Environ. Res. Lett..

[14] Pascal M., Wagner V., Corso M., Laaidi K., Ung A., Beaudeau P. (2018). Heat and cold related-mortality in 18 French cities. Environ. Int..

[15] Song B.-G., Park K.-H., Kim G.-A., Kim S.-H., Park G.-U., Mun H.-S. (2020). Analysis of Relationship between the Spatial Characteristics of the Elderly Population Distribution and Heat Wave based on GIS—Focused on Changwon City. J. Korean Assoc. Geogr. Inf. Stud..

[16] Pyrgou A., Santamouris M. (2020). Probability Risk of Heat- and Cold-Related Mortality to Temperature, Gender, and Age Using GAM Regression Analysis. Climate.

[17] (2021). The Lancet Health in a world of extreme heat. Lancet.

[18] Gobierno de España (2021). Plan Nacional de Actuaciones Preventivas de los Efectos del Exceso de Temperatura sobre la Salud. https://www.sanidad.gob.es/ciudadanos/saludAmbLaboral/planAltasTemp/2021/docs/Plan_Calor_2021.pdf.

[19] Hanna E.G., Tait P.W. (2015). Limitations to Thermoregulation and Acclimatization Challenge Human Adaptation to Global Warming. Int. J. Environ. Res. Public Health.

[20] Turek-Hankins L.L., Coughlan de Perez E., Scarpa G., Ruiz-Diaz R., Schwerdtle P.N., Joe E.T., Galappaththi E.K., French E.M., Austin S.E., Singh C. (2021). Climate change adaptation to extreme heat: A global systematic review of implemented action. Oxf. Open Clim. Chang..

[21] Åström D.O., Tornevi A., Ebi K.L., Rocklöv J., Forsberg B. (2016). Evolution of Minimum Mortality Temperature in Stockholm, Sweden, 1901–2009. Environ. Health Perspect..

[22] Sanchez Martinez G., De’Donato F., Kendrovski V., WHO Regional Office for Europe (2021). Heat and Health in the WHO European Region: Updated Evidence for Effective Prevention.

[23] Jonsson A.C., Lundgren L. (2015). Vulnerability and adaptation to heat in cities: Perspectives and perceptions of local adaptation decision-makers in Sweden. Local Environ..

[24] Díaz J., Carmona R., Mirón I., Ortiz C., Linares C. (2015). Comparison of the effects of extreme temperatures on daily mortality in Madrid (Spain), by age group: The need for a cold wave prevention plan. Environ. Res..

[25] Iñiguez C., Ballester F., Ferrandiz J., Pérez-Hoyos S., Sáez M., López A. (2010). Relation between Temperature and Mortality in Thirteen Spanish Cities. Int. J. Environ. Res. Public Health.

[26] Huang Y., Yang J., Chen J., Shi H., Lu X. (2022). Association between ambient temperature and age-specific mortality from the elderly: Epidemiological evidence from the Chinese prefecture with most serious aging. Environ. Res..

[27] Petkova E.P., Gasparrini A., Kinney P.L. (2014). Heat and mortality in New York City since the beginning of the 20th century. Epidemiology.

[28] Jiao Y., Yu H., Wang T., An Y., Yu Y. (2017). Thermal comfort and adaptation of the elderly in free-running environments in Shanghai, China. Build Environ..

[29] Instituto Nacional de Estadística Proporción de Personas Mayores de Cierta Edad por Provincia. https://www.ine.es/jaxiT3/Tabla.htm?t=1488.

[30] Pérez Díaz J., Abellán García A., Aceituno Nieto P., Ramiro Fariñas D. (2020). Un Perfil de las Personas Mayores en España 2020.

[31] Martínez-Solanas È., Basagaña X. (2019). Temporal changes in temperature-related mortality in Spain and effect of the implementation of a Heat Health Prevention Plan. Environ. Res..

[32] Boeckmann M., Rohn I. (2014). Is planned adaptation to heat reducing heat-related mortality and illness? A systematic review. BMC Public Health.

[33] Schifano P., Leone M., de Sario M., Dedonato F., Bargagli A.M., Dippoliti D., Marino C., Michelozzi P. (2012). Changes in the effects of heat on mortality among the elderly from 1998–2010: Results from a multicenter time series study in Italy. Environ. Health.

[34] Benmarhnia T., Bailey Z., Kaiser D., Auger N., King N., Kaufman J.S. (2016). A Difference-in-Differences Approach to Assess the Effect of a Heat Action Plan on Heat-Related Mortality, and Differences in Effectiveness According to Sex, Age, and Socioeconomic Status (Montreal, Quebec). Environ. Health Perspect..

[35] Barrett J.R. (2015). Increased Minimum Mortality Temperature in France: Data Suggest Humans Are Adapting to Climate Change. Environ. Health Perspect..

[36] Díaz J., Carmona R., Mirón I.J., Luna M.Y., Linares C. (2018). Time trend in the impact of heat waves on daily mortality in Spain for a period of over thirty years (1983–2013). Environ. Int..

[37] Li T., Horton R.M., Bader D.A., Zhou M., Liang X., Ban J., Sun Q., Kinney P.L. (2016). Aging Will Amplify the Heat-related Mortality Risk under a Changing Climate: Projection for the Elderly in Beijing, China. Sci. Rep..

[38] Botzen W.J.W., Martinius M.L., Bröde P., Folkerts M.A., Ignjacevic P., Estrada F., Harmsen C.N., Daanen H.A.M. (2020). Economic valuation of climate change–induced mortality: Age dependent cold and heat mortality in the Netherlands. Clim. Chang..

[39] Navas-Martín M.Á., López-Bueno J.A., Díaz J., Follos F., Vellón J.M., Mirón I.J., Luna Y., Sánchez-Martínez G., Culqui D., Linares C. (2022). Effects of Local Factors on Adaptation to Heat in Spain (1983–2018). Environ. Res..

[40] Morgenstern H. (1995). Ecologic studies in epidemiology: Concepts, principles, and methods. Annu. Rev. Public Health.

[41] Luo Q., Li S., Guo Y., Han X., Jaakkola J.J.K. (2019). A systematic review and meta-analysis of the association between daily mean temperature and mortality in China. Environ. Res..

[42] Aboubakri O., Khanjani N., Jahani Y., Bakhtiari B. (2019). Attributable risk of mortality associated with heat and heat waves: A time-series study in Kerman, Iran during 2005–2017. J. Biol..

[43] Lo Y.T.E., Mitchell D.M., Thompson R., O’Connell E., Gasparrini A. (2022). Estimating heat-related mortality in near real time for national heatwave plans. Environ. Res. Lett..

[44] Honda Y., Onozuka D. (2020). Heat-Related Mortality/Morbidity in East Asia. Extreme Weather Events and Human Health. https://link.springer.com/chapter/10.1007/978-3-030-23773-8_10.

[45] Masselot P., Ouarda T.B.M.J., Charron C., Campagna C., Lavigne É., St-Hilaire A., Chebana F., Valois P., Gosselin P. (2022). Heat-related mortality prediction using low-frequency climate oscillation indices: Case studies of the cities of Montréal and Québec, Canada. Environ. Epidemiol..

[46] Åström D.O., Veber T., Martinsone Ž., Kalužnaja D., Indermitte E., Oudin A., Orru H. (2019). Mortality Related to Cold Temperatures in Two Capitals of the Baltics: Tallinn and Riga. Medicina.

[47] Péres W.E., Ribeiro A.F.S., Russo A., Nunes B. (2020). The Association between Air Temperature and Mortality in Two Brazilian Health Regions. Climate.

[48] Todd N., Valleron A.J. (2015). Space-Time Covariation of Mortality with Temperature: A Systematic Study of Deaths in France, 1968–2009. Environ. Health Perspect.

[49] Hajat S., Armstrong B., Baccini M., Biggeri A., Bisanti L., Russo A., Paldy A., Menne B., Kosatsky T. (2006). Impact of high temperatures on mortality: Is there an added heat wave effect?. Epidemiology.

